# Developmental Cut-Points for Atypical Speech Intelligibility in Children With Cerebral Palsy

**DOI:** 10.1044/2022_JSLHR-22-00310

**Published:** 2023-03-09

**Authors:** Katherine C. Hustad, Tristan J. Mahr, Jennifer U. Soriano, Paul J. Rathouz

**Affiliations:** a Department of Communication Sciences and Disorders, University of Wisconsin–Madison; b Waisman Center, University of Wisconsin–Madison; c Department of Population Health, Dell Medical School, The University of Texas at Austin

## Abstract

**Purpose:**

Early identification of speech motor involvement (SMI) in children with cerebral palsy (CP) is difficult because of overlapping features with many aspects of typical speech development. Quantitative measures of speech intelligibility have the potential to differentiate between children with SMI and those with no SMI (NSMI). We examined thresholds for speech intelligibility development in children with CP relative to the low end of age-specific typical developmental expectations. We sought to determine whether there were intelligibility differences between children with CP and NSMI versus typically developing (TD) age-mates across the range of development and whether there were differences between children with CP who have NSMI and those with CP who have SMI across the range of development based on speech intelligibility.

**Method:**

We used two large existing data sets that included speech samples from children between the ages of 2.5 and 8 years. One data set included 511 longitudinal speech samples from children with CP; the other included 505 cross-sectional speech samples from TD children. We examined receiver operating characteristic curves and sensitivity/specificity results by age for differentiating among groups of children.

**Results:**

TD children versus those with CP and NSMI showed differentiation in their speech intelligibility across all ages, but the strength of differentiation was only marginally above chance. Children with CP and NSMI showed clear differentiation in their speech intelligibility from those with CP and SMI beginning at the earliest age point. Children with CP who have intelligibility below 40% at the age of 3 years have a very high probability of having SMI.

**Conclusions:**

Early intelligibility screening should be performed in children diagnosed with CP. Those with intelligibility below 40% at 3 years of age should be referred immediately for speech assessment and treatment.

Children with cerebral palsy (CP) are at risk for a range of communication-related challenges. Mei et al. (2020)  suggest that approximately 80% of children with CP have a speech disorder. More specifically, about 50% of children with CP are thought to have the speech motor disorder *dysarthria* (Nordberg et al., 2013). Dysarthria is almost always associated with reduced speech intelligibility (Yorkston et al., 1999), which, in turn, can impact functional communication, social participation, educational achievement, and quality of life (Dickinson et al., 2007). Interventions to maximize speech intelligibility are essential to reduce or prevent communication breakdown and/or failure.

Because children with CP vary considerably in their communication abilities, several different classification systems have been developed as a means of reducing heterogeneity. These classification systems were largely intended for epidemiological tracking purposes, and as such, classifications can be made through record review or caregiver report without a direct observation of the individual. However, there are important limitations to this approach, namely that underlying speech and language strengths and challenges are not directly quantified; thus, their contribution to overall communication ability is unknown. Research in our laboratory has used a data-based classification model for characterizing constellations of speech and language abilities in children with CP, based on direct measurement (Hustad et al., 2010). In its most basic form, this model has four categories or levels. These are as follows: children who have no speech motor involvement (NSMI) and appear otherwise typical in terms of their communication ability, children with speech motor involvement (SMI) who have language comprehension that is typical (SMI-LCT), children with SMI who have language comprehension impairment (SMI-LCI), and children who are unable to speak due to anarthria (ANAR). Our work suggests that these profiles are not clinically discernable until children are at about 4 years of age, primarily because the presence or absence of SMI is difficult to definitively determine in very young children. In great part, this is because features of typical speech development (and its wide range of variability) overlap considerably with features of SMI, more specifically dysarthria (Schölderle et al., 2020). In addition, children with CP may be delayed in the onset of speech production (Hustad et al., 2017) and, therefore, may lag behind age expectations for speech development. Although children with CP who have moderate or severe SMI often show clear clinical indications at early ages, others with milder involvement may be more difficult to differentiate from typical peers until they are older (Hustad et al., 2014).

Speech intelligibility measures may offer one way to differentiate between subsets of children with CP and their typically developing (TD) peers in order to identify children who lag behind the wide range of typical variability. Two recent large-scale sets of studies on speech intelligibility have revealed several key findings. First, in children with CP, studies suggest that early intelligibility development beginning at 2.5 years of age is highly predictive of later outcomes (Hustad et al., 2017; Hustad, Mahr, Broman, & Rathouz, 2020; Hustad, Sakash, Natzke, et al., 2019). In fact, we can predict with a good degree of certainty, based on intelligibility data at 3 years of age, what a child's intelligibility will be at 8 years of age if a child has CP and their speech and language classification is known (i.e., NSMI, SMI-LCT, SMI-LCI, or ANAR; Hustad, Mahr, Broman, & Rathouz, 2020). Furthermore, this research has shown that children with CP and SMI (for both SMI-LCT and SMI-LCI) lagged behind their counterparts who had CP and NSMI with regard to age of steepest intelligibility growth and intelligibility outcomes at 8 years of age (Mahr et al., 2020). Importantly, because these studies were longitudinal, classifications of children into profile groups (i.e., NSMI, SMI-LCI, or SMI-LCT) were made at 4–5 years of age, and data from earlier time points were classified retrospectively based on later emerging information. A key challenge that remains a barrier to treatment is early diagnosis of speech and language delays. To date, we have not been able to clearly differentiate children with CP into profile groups prospectively because of the aforementioned challenges with disentangling the impacts of neuromotor disorder from the impacts of development. Relatedly, a serious problem that has impeded our ability to interpret intelligibility findings, such as those described in our previous studies of children with CP (Hustad et al., 2017; Hustad, Mahr, Broman, & Rathouz, 2020; Hustad, Sakash, Natzke, et al., 2019), relative to age-level expectations has been a lack of normative data for TD children. If the range of typical developmental expectation is not well understood, reliable identification of children who fall outside of that range is limited.

Recently, large-scale studies on typical development using metrics that are clinically important for children at risk for SMI have begun to establish norms that can be used for determining whether or not children fall outside the full range of typical age expectations. These studies have established speech intelligibility growth curves, including ranges of variability according to targeted percentile distributions for children between 2 and 10 years of age (Hustad, Mahr, Natzke, & Rathouz, 2020; Hustad et al., 2021; Mahr et al., 2021). Results have demonstrated that intelligibility in TD children grows rapidly in the preschool years and that the range of typical variability is very wide. Furthermore, TD children experience their peak intelligibility growth rate prior to 3 years of age; however, intelligibility continues to improve with age throughout middle childhood, and variability across children decreases with age. Although results from these normative studies have provided much-needed information, the next step is to examine normative data across the full range of performance quantiles with parallel data from children with CP in order to determine the ways in which subgroups of children can be differentiated on the basis of speech intelligibility scores.

In this study, we employed data from two previous projects: one that examined longitudinal development of intelligibility in children with CP (Mahr et al., 2020) and one involving the creation of growth curves for speech intelligibility development in TD children (Hustad et al., 2021). In this study, we jointly model the two unique data sets to address novel questions. Because reduced speech intelligibility is a ubiquitous feature of dysarthria, we expect that by comparing the performance of children with CP and SMI versus that of children with CP and NSMI (as determined retrospectively based on longitudinal outcome data), we may be able to identify cut-points or thresholds that indicate the presence of SMI prior to the age of 4 years. Similarly, preliminary research has suggested that there may be subtle but significant differences between TD children and those with CP and NSMI at 5 years of age (Hustad, Sakash, Broman, & Rathouz, 2019), but the extent to which such a difference exists at earlier and later ages is unknown. The ability to make these differentiations may have important consequences for intervention and later outcomes. In particular, the identification of SMI at the earliest possible age may lead to earlier speech intervention and/or treatment focused on early augmentative and alternative communication (AAC) systems and strategies. Furthermore, age-specific performance cut-points or benchmarks could serve as useful clinical indicators of both severity and the need for intervention. Our goal in this study was to examine thresholds for speech intelligibility development in children with CP, relative to the low end of age-specific typical developmental expectations. Specifically, we sought to determine whether children with CP and NSMI were clinically differentiable from TD age-mates across the range of development. We also sought to determine whether children with CP and NSMI were clinically differentiable from children with CP and SMI early in development based on speech intelligibility. Toward this end, we asked the following research questions:

1. Can speech intelligibility measures differentiate between children with CP who have NSMI versus TD children? If so, what are the intelligibility thresholds associated with .90 specificity by age? We use a specificity of .90 because we sought to use the 10th percentile of typical development as a cutoff for delayed- or disordered-speech performance. Thus, children whose performance is below the 10th percentile of typical development will be detected.
2. Can speech intelligibility measures differentiate between children with CP who have SMI versus children with CP who have NSMI? If so, what are the intelligibility thresholds associated with .90 sensitivity by age? We examine sensitivity here rather than specificity (as in Research Question 1) because all of the children in this comparison have CP, so the important clinical task is to detect whether a child has speech motor impairment (positive case). Thresholds that target .90 sensitivity cast a wide net, so to speak, so that 90% of children with CP who have SMI can be detected.

On the basis of our previous research (Hustad et al., 2012), we hypothesized that TD children would show differentiation in their speech intelligibility from those with CP and NSMI. We also expected that differentiation might reduce with age based on our normative work showing that TD children tend to become more homogeneous and less variable at older ages as they advance closer to the ceiling of intelligibility development (Hustad et al., 2021). Regarding differences between children with CP and NSMI versus those with CP and SMI, we expected clear differentiation at the earliest age, with increasing differentiation over the course of development, again based on our previous work showing that variability increased among children with SMI over time (Mahr et al., 2020).

## Method

This study was approved by the University of Wisconsin–Madison Social and Behavioral Sciences Institutional Review Board (No. 2018–0580). Written informed consent or assent was provided on behalf of or by all participants.

### Participants

#### Children With CP

A total of 65 children with CP (34 boys, 31 girls) were included in this study. Data from these children were reported in a longitudinal study of speech intelligibility development by Mahr et al. (2020). We used this original sample minus two observations in which the children fell outside the 30- to 96-month age range; the remaining sample size was 511 longitudinal data points. Each child provided between two and 11 speech samples, with an average of 7.9 samples (*SD* = 2.2) per child. Speech samples were collected at 6-month intervals through 8 years of age. All children were from homes where American English was the primary language. Demographic information including CP diagnosis is presented in Table 1.

**Table 1.** Demographic characteristics of participants with cerebral palsy (CP).

Variable	CP (*N* = 65)
Ratio, male:female	34:31
GMFCS	
I	32
II	18
III	3
IV	7
V	3
(Missing)	2
CP type	
Spastic	
Diplegia	13
Hemiplegia (left)	14
Hemiplegia (right)	14
Triplegia	2
Quadriplegia	6
Unknown	1
Dyskinetic	1
Ataxic	5
Mixed	1
Hypotonic	1
Unknown	7

*Note.* Please see Hustad, Mahr, Broman, and Rathouz (2020); Hustad, Sakash, Natzke, et al. (2019); and Mahr et al. (2020) for additional demographic information about this longitudinal cohort of children with CP. GMFCS = Gross Motor Function Classification System.

#### Children with TD

A total of 505 TD children provided cross-sectional speech samples between the ages of 30 and 96 months (238 boys, 267 girls) for this study. These children are an age-based subset of the sample of 30- to 119-month-old children reported in a cross-sectional study of developmental growth curves for speech intelligibility by Hustad et al. (2021) and for articulation rate by Mahr et al. (2021). All children passed speech, language, and hearing screening measures and had no history of developmental delay. See previous papers for methodological details regarding sampling and participant screening (Hustad, Mahr, Natzke, & Rathouz, 2020; Hustad et al., 2021). In this study, we analyze intelligibility data for children with CP and TD children in a novel way to address different questions from those in our previous publications. See Table 2 for age and sex distributions of TD children.

**Table 2.** Age and sex distribution of the cross-sectional sample of typically developing children by 12-month age bin.

Age range (months)	*N* Children	*n* Boys	*n* Girls
30–41	107	52	55
42–53	111	38	73
54–65	110	59	51
66–77	96	52	44
78–96	81	37	44

*Note.* Children above the age of 78 months were pooled into one bin because of developmental homogeneity in performance.

#### Adult Listeners

A total of 2,034 normal-hearing adults served as listeners in this study (521 men, 1,510 women; three declined responses). The mean age was 21.2 years (*SD* = 3.8). Two different listeners heard the speech of each child at each visit; each listener heard one child producing all stimulus material in random order. Listeners were recruited from the local community; reported no more than incidental experience listening to or communicating with persons having communication disorders; and reported a negative history of language, learning, or cognitive disability. Listeners were compensated monetarily for their participation. Listener data for speech intelligibility were reported in previous papers (Hustad, Mahr, Natzke, & Rathouz, 2020; Hustad et al., 2021).

### Materials and Procedure

#### Speech Intelligibility

All children produced the same corpus of speech stimuli, which were 60 different multiword utterances from the Test of Children's Speech (TOCS+; Hodge et al., 2007) following a recorded model. Utterances varied in length from two to seven words, with 10 items per length. A list of stimulus utterances is available as supplemental material in Hustad et al. (2021). Speech samples from children were recorded using a digital audio recorder (Marantz PMD570) at a 44.1-kHz sampling rate (16-bit quantization), with a condenser studio microphone (Audio-Technica AT4040) positioned next to each child approximately 18 in. from the child's mouth. Additional information regarding materials and methods is presented in earlier papers (Hustad, Mahr, Broman, & Rathouz, 2020; Hustad et al., 2021). In this study, we report intelligibility for multiword stimuli for children with CP and TD children.

Listeners made orthographic transcriptions in a sound-attenuating suite of all stimulus material produced by the child. The presentation level of speech samples was calibrated to a peak level of approximately 75 dB SPL for each individual child to ensure some uniformity of presentation loudness among listeners of different children. The presentation order was randomized for each listener. Orthographic transcripts generated by listeners were scored by counting the number of words that were an exact phonemic match to the target word produced by the child for each listener. The total number of words transcribed correctly by each of the two listeners per child was averaged and then divided by the number of words produced by each child to yield a mean intelligibility score expressed as a proportion for each child and each visit.

We used an intraclass correlation coefficient (ICC) to calculate reliability of listeners, estimated using the irr R package (Version 0.84.1; Gamer et al., 2019). We used an average-score, consistency-based, one-way random-effects model (with the random factor being child-level intercepts). For the TD group, we found a strong agreement for the 505 listener pairs, ICC(2) = .982, 95% CI [.978, .984], and for the CP group, we found a strong agreement for the 511 listener pairs, ICC(2) = .987, 95% CI [.985, .989]. The average difference between the two listeners of each child for each visit was 3.7 percentage points (*SD* = 3.6) for the TD group and 5.0 percentage points (*SD* = 4.7) for the CP group.

#### Classification Into Profile Groups

Children were classified into one of two speech-language profile groups following our earlier work (Hustad et al., 2010). Briefly, each child was classified based on clinical judgment of the presence or the absence of SMI by two speech-language pathologists. Children with CP were classified as having NSMI if they had no evidence of speech impairment based on clinician observation during the data collection session, which was confirmed via a review of video and audio recordings after the session. Children were classified as having SMI through clinical observation of the presence or absence of dysarthria and/or apraxia features, which collectively included, but were not limited to, facial asymmetry; drooling; hypernasality; short breath groups; breathy, harsh, or wet vocal quality; imprecise articulation; and consonant or vowel substitutions, distortions, or omissions that were not age appropriate. Perceptual judgments were made during administration of the TOCS+ and during a spontaneous speech sample. Classification agreement between the two speech-language pathologist raters was 100%.

Classification methods have been described in detail previously (Hustad, Mahr, Broman, & Rathouz, 2020). In our other studies, we have further classified children based on the presence or the absence of language comprehension impairment; however, this study focuses only on speech performance, and further subdividing children with CP would reduce statistical precision. Thus, we examined only two speech-based groupings of children with CP.

### Statistical Analyses

**Figure 1.**
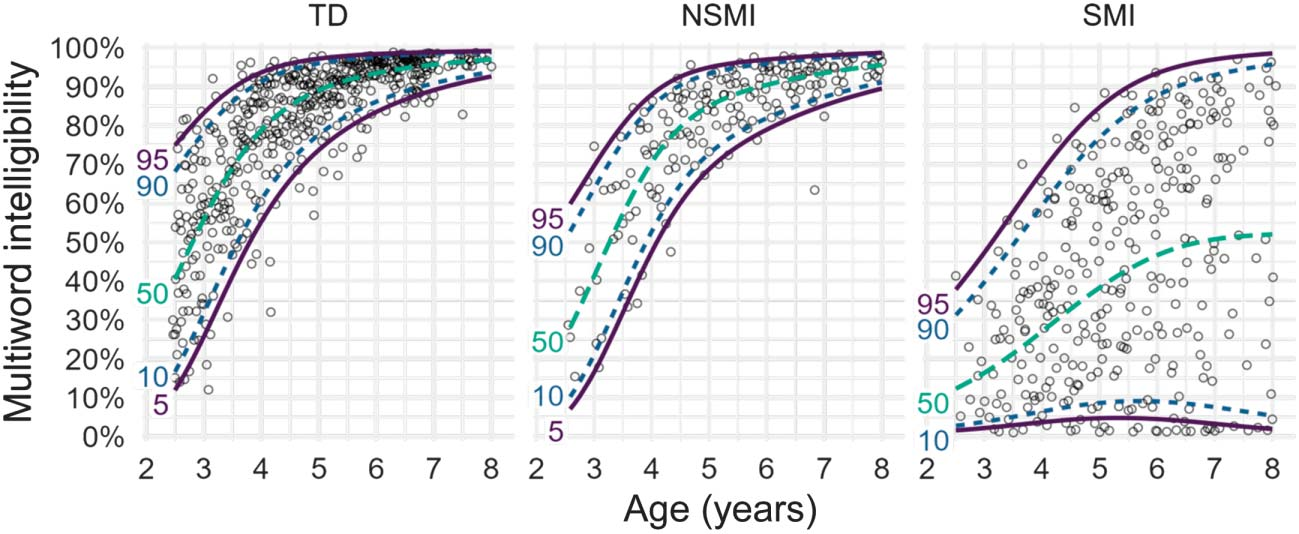
Percentile growth curves for typically developing (TD) children (left panel), children with cerebral palsy and no speech motor involvement (NSMI; middle panel), and children with cerebral palsy and speech motor involvement (SMI; right panel). Lines were estimated using beta regression with natural cubic splines, so that the average intelligibility and the variability around that average (i.e., percentiles) could flexibly change with age. Lines represent the 95th, 90th, 50th, 10th, and 5th percentiles in each panel.

In Hustad et al. (2021), we computed flexible percentile (quantile) growth curves for the intelligibility of TD children. The approach incorporated imputation and weighting techniques to account for utterance lengths with missing items. Here, we applied these same analytic approaches to the sample of children with CP, divided into two groups: those with NSMI and those with SMI (see Figure 1). Briefly, we fitted three separate (TD, SMI, and NSMI) flexible beta regression models to the data to estimate how the full distribution of intelligibility scores (rather than just the average intelligibility value) changes with age, allowing us to estimate separate developmental trajectories for the 5th, 10th, and other intelligibility percentiles. These models used a three-degree-of-freedom natural cubic spline for the mean intelligibility value and a two-degree-of-freedom natural cubic spline for the precision of the intelligibility scores. That is, we model the effect of age in a piecewise manner. The mean model's three degrees of freedom indicates that age range was divided into tertiles (three bins containing equal numbers of observations), and each bin had a basis function associated with it. Thus, *local* age effects could be modeled. Adjacent basis functions also overlapped so that the change from bin to bin would be smooth. From the fitted age-specific distributions, we extracted intelligibility density functions, by age, in each group and used these to derive receiver operating characteristic (ROC) statistics. To estimate statistical uncertainty, we repeated this modeling and derivative calculations on 2,000 bootstrap resamples. We performed stratified bootstrap resampling for the TD group by splitting children into five age strata (2;6–3;5 [years;months], 3;6–4;5, 4;6–5;5, 5;6–6;5, and 6;6–8;0). For the children in the NSMI and SMI groups, who provided longitudinal data, we resampled whole children as opposed to individual intelligibility values.

For Research Question 1, we examine the 10th percentile of the TD group as a benchmark for a possible intelligibility deficit or delay (i.e., a *specificity* of .90). For Research Question 2, we examine what level of intelligibility will detect 90% of children with CP who have speech motor impairment (i.e., a *sensitivity* of .90). More specifically, for Research Question 1, we treated the TD and NSMI groups as reference (control) and affected (case) groups, respectively. We then overlaid the fitted NSMI distribution onto the fitted 10th percentile curve from the TD panel. By definition, 90% of TD children will fall above this 10th percentile curve, so this curve would have a specificity of .90. The fitted percentage of children with NSMI below the curve represents the sensitivity of this intelligibility threshold. For Research Question 2, the NSMI and SMI groups are the reference and affected groups, respectively. Here, we overlay the NSMI distribution onto the fitted 90th percentile curve from the SMI group. Because 90% of children with SMI will fall below this curve, it has a sensitivity of .90 for detecting SMI within the CP population. The percentage of children with NSMI above the curve represents, in this case, the specificity of this threshold. In each analysis, we obtain threshold curves, sensitivities, specificities, and areas under the ROC curve (ROC-AUCs) by age. A global metric for evaluating the performance of a classifier or diagnostic tool, the ROC-AUC is the probability that a diagnostic tool will correctly sort a pair of children of the same age into affected and reference groups.

We used the R programming language (Version 4.2.0; R Core Team, 2021) for our analysis. We used Generalized Additive Models for Location, Scale and Shape (GAMLSS; Version 5.4.3; Rigby & Stasinopoulos, 2005) for beta regression and pROC (Version 1.18.0; Robin et al., 2011) for ROC statistics.

## Results

Figure 1 shows percentile growth curves for intelligibility for each of the three groups of children. For the TD and NSMI groups, average intelligibility increased with age, and variability in intelligibility decreased with age. This is observed in the narrowing range between the top and bottom percentile lines for higher ages. For the SMI group, average intelligibility also increased with age (as seen in the 50th percentile line), but variability increased, as observed in the widening gap between the top and bottom percentile lines.

### Question 1: NSMI Versus TD

**Figure 2.**
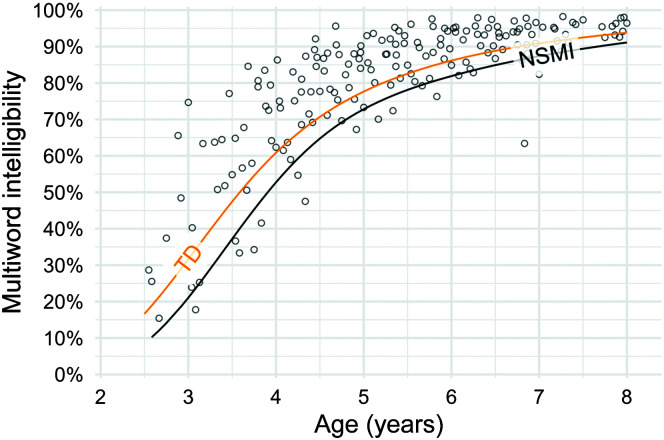
Comparison of the 10th percentile lines for children in the TD group and children with cerebral palsy in the NSMI group. Points represent children with cerebral palsy and NSMI. Visually, the two percentile lines are parallel. The average horizontal distance between the lines was 6 months. NSMI = children with cerebral palsy who have no speech motor involvement; TD = typically developing children.

**Figure 3.**
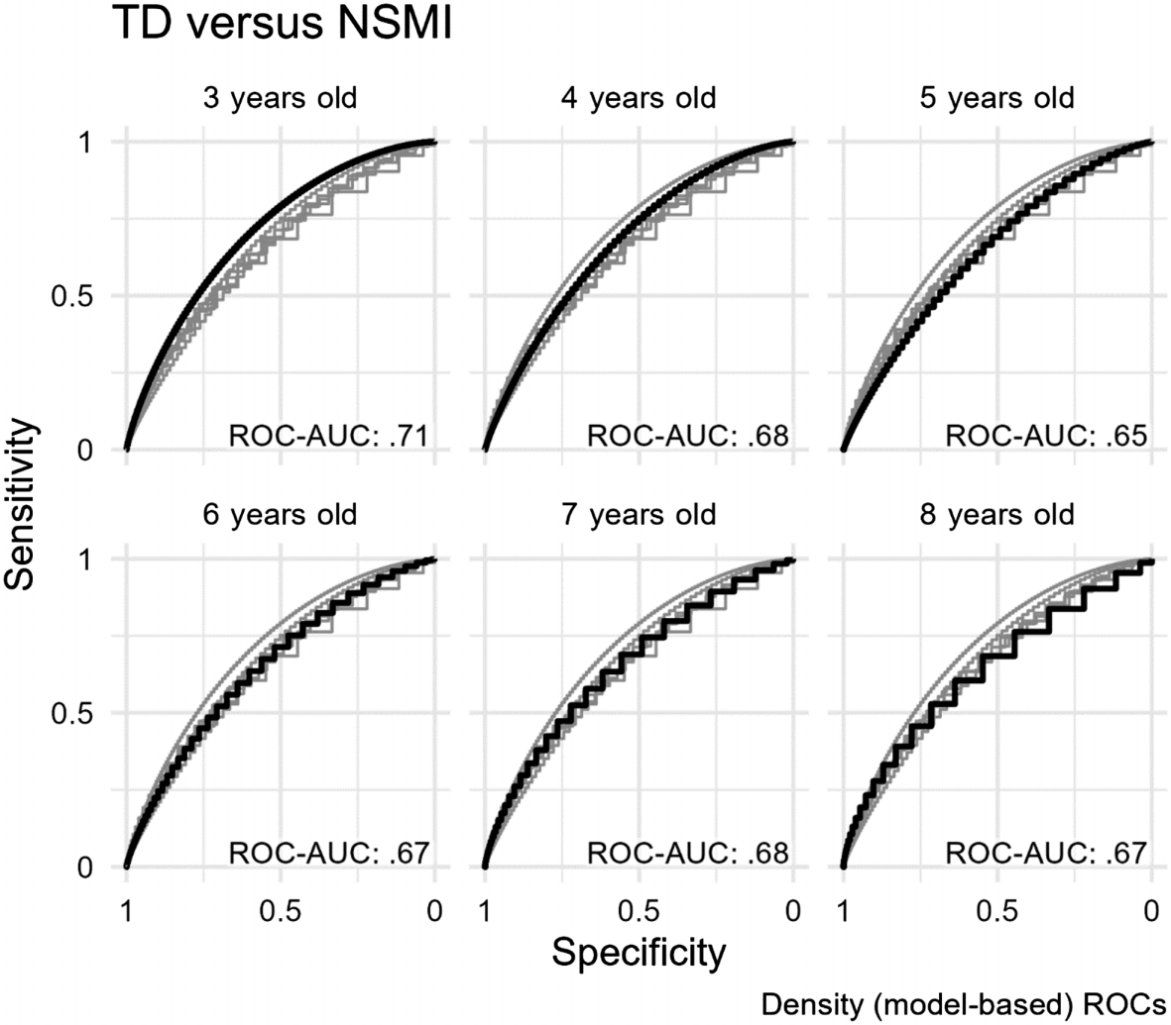
Receiver operating characteristic (ROC) curves for the differentiation of children in the TD group and children with cerebral palsy in the NSMI group. Panels are separated by age, with the dark line showing the ROC curve for the specific age panel and the light lines showing the ROC curves for the other age panels. NSMI = children with cerebral palsy who have no speech motor involvement; TD = typically developing children; ROC-AUC = area under the ROC curve.

Figure 2 shows intelligibility performance for children in the NSMI group, with the 10th percentile line for the TD and NSMI groups overlaid. The two 10th percentile lines are essentially parallel, and the average horizontal distance between the lines was 6 months, 95% bootstrap CI [2, 10], indicating that the NSMI group lags behind the TD group by 6 months. A similar comparison for the 50th percentile lines shows an average distance of 8 months, 95% CI [4, 12]. Thus, the NSMI group tends to achieve the same intelligibility outcomes as the TD group but with a 6- to 8-month delay. Figure 3 shows density (model-estimated) ROC curves by age. On the basis of ROC-AUC statistics, the TD and NSMI groups were only marginally differentiable at all ages. At the age of 3 years, the model-based ROC-AUC statistic was .71, 95% bootstrap CI [.58, .86]. The ROC-AUC value decreased to .68, 95% bootstrap CI [.57, .78], at the age of 4 years and remained similar at later ages. Note that random classification of children—that is, randomly assigning them the labels “TD” or “NSMI”—would yield an ROC-AUC value of .5; thus, values in the range of .6–.7 may not represent an important clinical difference between groups.

Table 3 shows intelligibility thresholds for the specificity of .90 (equivalent to the 10th percentile of the TD group) and associated sensitivities by age. Applying a threshold with .90 specificity is akin to flagging children who fall below the 10th percentile in TD intelligibility as delayed or requiring further evaluation. Results indicate that intelligibility thresholds at the ages of 3, 4, 5, 6, 7, and 8 years are *at* the following percentages for TD children: 31%, 60%, 77%, 86%, 90%, and 93%, respectively. Because the NSMI percentile lagged behind the TD percentile by about 6–8 months, the proportion of children in the NSMI group falling under the TD percentile line was expected to be small. Indeed, the sensitivity of this threshold was between .21 and .29.

**Table 3.** Intelligibility thresholds required to differentiate children in the TD group and children with cerebral palsy in the NSMI group with .90 specificity.

Comparison	Age (years)	Intelligibility threshold (10th percentile TD)	Sensitivity	Specificity (fixed to .90)
TD versus NSMI	3	31.5%	.288	.899
	4	60.5%	.235	.901
	5	77.5%	.211	.898
	6	86.0%	.247	.897
	7	90.5%	.262	.905
	8	93.5%	.279	.903

*Note.* The TD group's 10th percentile showed poor sensitivity for detecting children in the NSMI group. TD = typically developing children; NSMI = children with cerebral palsy who have no speech motor involvement.

### Question 2: SMI Versus NSMI

**Figure 4.**
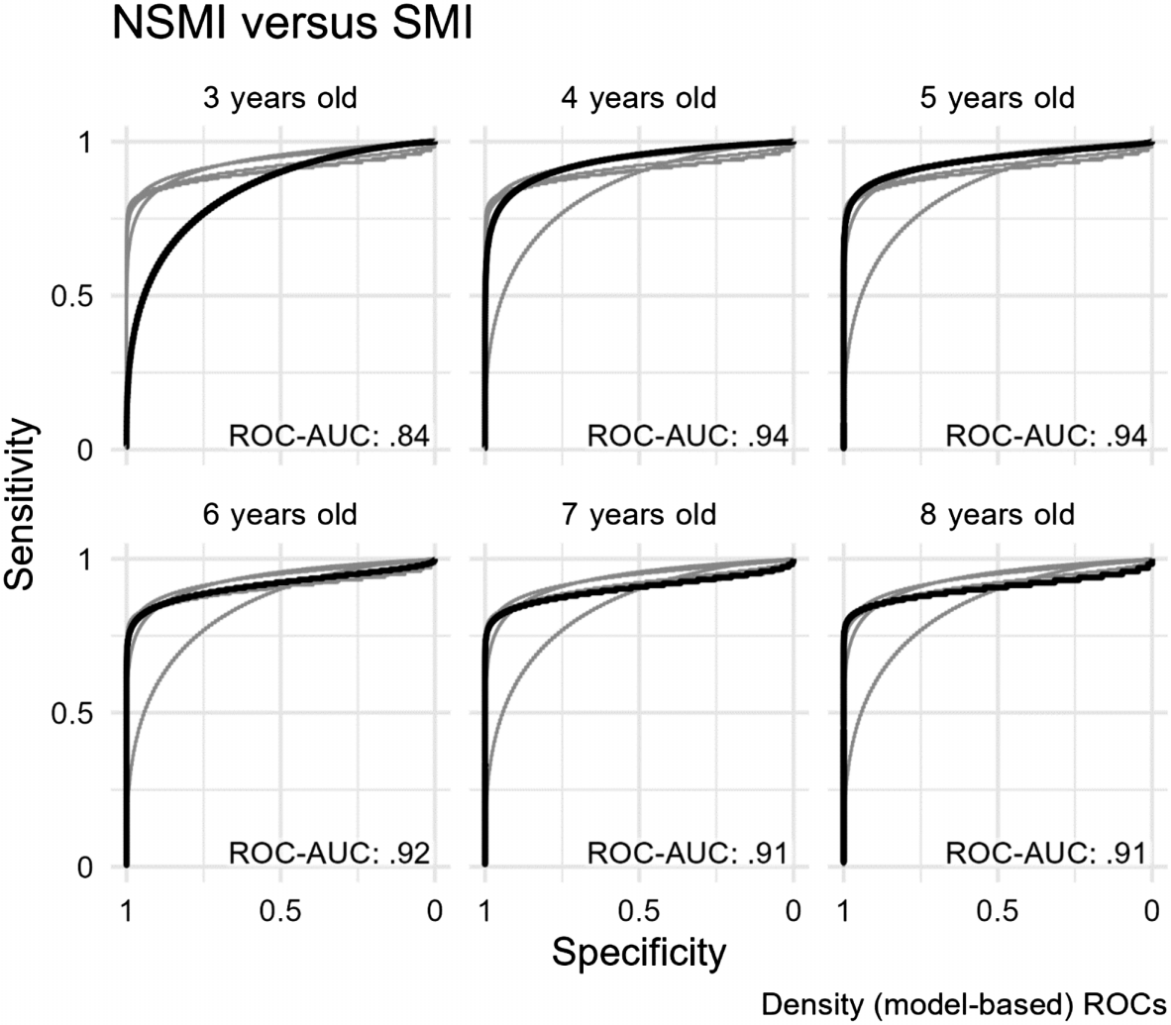
Receiver operating characteristic (ROC) curves for the differentiation of children with cerebral palsy in the NSMI versus SMI groups. Panels are separated by age, with the dark line showing the ROC curve for the specific age panel and the light lines showing the ROC curves for the other age panels. NSMI = children with cerebral palsy who have no speech motor involvement; SMI = children with cerebral palsy who have speech motor involvement; ROC-AUC = area under the ROC curve.

Figure 4 shows density ROC curves by age for SMI and NSMI groups. On the basis of ROC-AUC statistics, the SMI and NSMI groups were clearly differentiable at all ages. At the age of 3 years, the model-based ROC-AUC statistic was .84, 95% bootstrap CI [.69, .95]. The ROC-AUC value increased to .94, 95% bootstrap CI [.89, .97], at the age of 4 years and remained similar at later ages.

Table 4 shows intelligibility thresholds for the sensitivity of .90 and associated specificities, along with positive predictive values (PPVs) by age. Note that a sensitivity of .9 is the intelligibility threshold below which 90% of children with SMI perform at a given age (i.e., the 90th percentile of the SMI group). Results indicate that intelligibility thresholds at the ages of 3, 4, 5, 6, 7, and 8 years are *at* the following percentages for children with SMI: 41%, 60%, 77%, 88%, 93%, and 95%, respectively. We note that these thresholds are very similar to those reported for TD children versus children with NSMI, with the exception of the threshold for 3-year-old children, which is about 10 percentage points higher when differentiating the SMI group from the NSMI group. An intelligibility threshold with a sensitivity of .9 indicates that a child with SMI has a .9 probability of having an intelligibility below that value. In diagnostic testing, the inverse probability [Prob(SMI *given* intelligibility < threshold)] is sometimes of greater interest and indicates the probability that a child with CP has SMI given that they scored below the threshold. This value is called the PPV, and its calculation requires accounting for the prevalence of SMI in the CP group. We use a prevalence of .667 based on our sample, which has 44 and 21 children in the SMI and NSMI groups, respectively. PPVs ranged from a minimum of .79 at the age of 3 years to a maximum of .91 at the age of 5 years. Thus, these intelligibility thresholds are highly predictive of whether or not a child with CP will have SMI.

**Table 4.** Intelligibility thresholds required to differentiate children with cerebral palsy in the NSMI versus SMI groups with .90 sensitivity.

Comparison	Age (years)	Intelligibility threshold (90th percentile SMI)	Sensitivity (fixed to .90)	Specificity	Prevalence	PPV
SMI versus NSMI	3	40.5%	.900	.511	.677	.794
	4	59.5%	.900	.786	.677	.898
	5	76.5%	.899	.817	.677	.911
	6	87.5%	.901	.676	.677	.854
	7	92.5%	.903	.576	.677	.817
	8	94.5%	.900	.609	.677	.828

*Note.* The NSMI group's 90th percentile intelligibility scores showed very strong positive predictive values (PPVs) for identifying children with SMI. SMI = children with cerebral palsy who have speech motor involvement; NSMI = children with cerebral palsy who have no speech motor involvement.

## Discussion

In this study, we examined whether speech intelligibility scores could differentiate between three groups of children: those with CP and NSMI, those with CP and SMI, and those with typical development. We were interested in the differentiation between children with CP and NSMI versus TD children because our preliminary work has suggested that children with CP and NSMI may lag behind TD peers in their intelligibility. We wondered if this finding would hold across a range of ages and with a large sample or whether any differentiation would reduce with age as children reach the ceiling of intelligibility development. We were also particularly interested in the extent to which intelligibility scores differentiated children with CP and SMI from those with CP and NSMI, especially at very young ages. Although children in these two groups become readily clinically differentiable later in the preschool years, we sought to determine whether they could be differentiated at earlier ages and, if so, what would the clinical cut-point for identification of SMI be in terms of speech intelligibility. To address these questions, we jointly modeled data from two data sets used in previous studies (Hustad et al., 2021; Mahr et al., 2020) and examined ROC curves. Key findings from this study were as follows. Children with CP and NSMI lagged slightly behind their TD peers in speech intelligibility from 2.5 to 8.0 years of age. However, children with CP and SMI showed clear differentiation from those with CP and NSMI, even at the earliest ages. These findings are discussed in detail below.

Intelligibility scores provided strong differentiation between children with SMI and those with NSMI, even as young as an average age of 3 years. Results showed that ROC-AUC values were very high at all ages (see Figure 4), indicating that speech intelligibility scores alone successfully classified children into binary groups—in this case, SMI versus NSMI—with a high degree of accuracy. However, the AUC value was slightly lower at 3 years of age than at older ages, likely owing to the large range of performance variability that has been documented in both TD children (Hustad et al., 2021) and children with CP (Mahr et al., 2020) in early development. It is noteworthy that an intelligibility score below 40% at 3 years of age had a PPV of .8, indicating an 80% probability of having SMI. This finding may provide a critical clinical threshold that can be used to prospectively identify children with CP who are most likely to have SMI and to begin to provide early speech and communication treatment to these children.

Another important finding from this study was that the strength of differentiation between children with CP and NSMI versus those with CP and SMI increased with age and then remained relatively stable through development, with a PPV of .9 by the age of 4 years. We highlight here that our results suggest that children with a known diagnosis of CP and who have multiword speech intelligibility below 60% at 4 years of age, below 77% at 5 years of age, below 88% at 6 years of age, below 93% at 7 years of age, and below 95% at 8 years of age have probabilities between 82% and 91% of having SMI. At older ages (e.g., above 5 years), the identification of SMI is generally relatively straightforward via a clinical examination, so these thresholds may be less powerful for identifying cases of SMI in older children with CP. However, for younger children, a general clinical guideline suggested by these results may be 40%, 60%, and 80% for ages 3, 4, and 5 years, respectively. That is, children with CP who have intelligibility below 40% at 3 years of age, below 60% at 4 years of age, and below 80% at 5 years of age are very likely to have SMI.

TD children and those with CP and NSMI showed some differentiation in their speech intelligibility development, although it was only marginally above chance. Children with CP and NSMI were slightly behind their TD peers in speech intelligibility over the full range of ages examined in this study. In developmental terms, this lag was equivalent to a delay of 6–8 months (see Figure 2). However, children with CP and NSMI followed the same pattern of development as TD children. Furthermore, the absolute difference between children in each group ranged from about 10% at earlier ages to about 4% at later ages. These findings are consistent with earlier work examining 5-year-old children (Hustad, Sakash, Broman, & Rathouz, 2019). At the age of 8 years, both groups of children still showed intelligibility growth. The extent to which the groups will further converge with age is unknown, but future research should examine this.

### Clinical Implications

All children with CP are at elevated risk of speech and language delay/disorder. In this study, we provide the first set of clinical decision-making guidelines based on normative intelligibility data showing that children with CP and SMI can be differentiated from those with CP and NSMI as early as 3 years of age. Thus, this study provides important information that could be useful for early identification of children with CP who have SMI. Our findings suggest that if a child has a diagnosis of CP, is between 2.5 and 3.5 years of age, and has multiword utterance intelligibility below 40%, that child has a very high probability of having SMI. Intelligibility measures reported in this article can be obtained for clinical use but are somewhat cumbersome because they require at least one listener unfamiliar with the child to make orthographic intelligibility transcriptions. This type of measure has been routinely used in clinical practice with adults who have motor speech disorders since the 1980s when the Assessment of Intelligibility of Dysarthric Speech (Yorkston et al., 1984) was introduced and then later via the Sentence Intelligibility Test (Yorkston et al., 1996). The TOCS+ (Hodge et al., 2007) sentences used in this article and in our previous work are available as supplemental material in our open access paper (Hustad et al., 2021). Measurement to determine whether a child's utterances are below 40% intelligible, at present, would involve the use of procedures similar to those presented here. Strategies for objectively measuring intelligibility more efficiently in clinical settings are needed so that findings such as those from this study can be readily applied in clinical assessment.

Early indicators of SMI should trigger immediate speech and language assessment and intervention. Along with speech motor–focused treatment, AAC interventions should be strongly considered in early childhood for children with CP and SMI to ensure access to expressive language modalities and for the advancement of social participation and functional communication. Many children with CP and SMI will have significantly reduced intelligibility throughout life and will therefore have long-term needs for AAC supports. We emphasize that AAC interventions and speech interventions are not mutually exclusive. AAC strategies and supports used in conjunction with natural speech can provide an important means to increase the intelligibility of speech to enhance functional communication (Hustad & Lee, 2008; Hustad et al., 2002). AAC can play different roles for supporting communication, depending on the context and on the communication partner. These roles include as a backup strategy to repair spoken communication breakdown, as a support for speech, as an equal partner with speech, or as a comprehensive replacement for speech (Hustad & Miles, 2010). Learning to use a range of multimodal tools (including speech along with AAC systems and strategies) at an early age is an important way to ensure the development of functional communication abilities.

Results of this study indicate that children with CP and NSMI, although differentiable from TD age-mates, have strong speech outcomes. For many children with CP and NSMI, speech intelligibility lags slightly behind age expectations, but data suggest that this gap reduces over time and may continue to close after the age of 8 years. These findings present a positive picture for speech development in children with CP and NSMI.

### Limitations and Future Directions

In this study, we examined speech intelligibility measures as obtained through elicited speech samples with naïve listeners who made orthographic transcriptions. Intelligibility data for all children, namely, those with CP and those with TD, were collected in a highly controlled laboratory setting and, thus, represent an idealized situation. As a result, some caution should be used when generalizing findings clinically. Specifically, currently, there are no *clinically efficient tools* to obtain objective intelligibility scores. Measurement of intelligibility requires orthographic transcription by an unfamiliar listener of elicited utterances produced by a child. The development and standardization of assessment items and a procedure for obtaining comparable intelligibility scores more efficiently are necessary to ensure that results from this study can be readily applied in clinical practice. Future research using different types of listening and speaking environments and different measures of speech intelligibility that are obtained in real-world settings should be investigated so that results can be directly translated. Furthermore, indirect measures of speech intelligibility, including rating scales and questionnaires, should be examined to determine if they provide the type of differentiation among children that the intelligibility measures examined in this study have shown.

## Data Availability Statement

Data supporting the analyses and results presented in this article may be made available from the authors on request. Data are not publicly available due to institutional review board protections.
